# Supplementary material for: Identification and functional analysis of non-coding regulatory small RNA FenSr3 in Bacillus amyloliquefaciens LPB-18
Source: PeerJ. 2023 May 15;11:e15236. doi: 10.7717/peerj.15236 (PMC10194069; doi:10.7717/peerj.15236)
Supplement: Supplemental Information 4 [file peerj-11-15236-s004.zip › KO/CK-vs-T1_map/map04932.html]

KEGG PATHWAY: Non-alcoholic fatty liver disease (NAFLD) - Reference pathway


|  |  |
| --- | --- |
| **Non-alcoholic fatty liver disease (NAFLD) - Reference pathway** |  |

[
Pathway menu
| Organism menu
| Pathway entry
| Show description
| User data mapping
]

Non-alcoholic fatty liver disease (NAFLD) represents a spectrum ranging from simple steatosis to more severe steatohepatitis with hepatic inflammation and fibrosis, known as nonalcoholic steatohepatitis (NASH). NASH may further lead to cirrhosis and hepatocellular carcinoma (HCC). This map shows a stage-dependent progression of NAFLD. In the first stage of NAFLD, excess lipid accumulation has been demonstrated. The main cause is the induction of insulin resistance, which leads to a defect in insulin suppression of free fatty acids (FAAs) disposal. In addition, two transcription factors, SREBP-1c and PPAR-alpha, activate key enzymes of lipogenesis and increase the synthesis of FAAs in liver. In the second stage, as a consequence of the progression to NASH, the production of reactive oxygen species (ROS) is enhanced due to oxidation stress through mitochondrial beta-oxidation of fatty acids and endoplamic reticulum (ER) stress, leading to lipid peroxidation. The lipid peroxidation can further cause the production of cytokines (Fas ligand, TNF-alpha, IL-8 and TGF), promoting cell death, inflammation and fibrosis. The activation of JNK, which is induced by ER stress, TNF-alpha and FAAs, is also associated with NAFLD progression. Increased JNK promotes cytokine production and initiation of HCC.

|  |  |  |
| --- | --- | --- |
| Reference pathway | 184% 150% 122% 100% 82% 67% 55% | 图片下载 |
